# Annotations

**Published:** 1900-01-20

**Authors:** 


### Annotations.

### Soldiers in Civilian Hospitals.

The suggestion that the various general and other hospitals should be prepared to receive the overflow from the military hospitals is an admirable one, and the response made from some of them shows how widespread is the desire to lend a helping hand to our sick and wounded on their return from the front, and to do all that is possible to lighten the task with which the Government will possibly be confronted should their number become considerable. If the matter can be so arranged as to eventuate in the using up of beds which are at present idle, of which there are many, everything is to be said in favour of the scheme. There are hospitals on every side whose habit it is never to be full, and these can well afford to fill their beds with soldiers; then, here and there, new hospitals may happen to be almost ready for opening in the place of old ones, as at Paisley, and where such is the case no special hardship to the poor will result by putting off the "flitting" for a few months, and giving the new wards to the soldiers for the time; and there are no doubt a few hospitals which having originally been built on too lavish a scale actually have wards, and perhaps wings, entirely unused, and these could, of course, be handed over to the authorities. It is to be feared, however, that some hospital committees, swayed by fashion or carried away by that wave of sympathy with brave but unfortunate soldiers which is now sweeping over the land, are offering what is really hardly theirs to give. It may, indeed, be suggested, with all deference to popular feeling, that in some cases, in undertaking to receive sick soldiers, committees are doing so not at the cost of their funds or their subscriptions, but at the cost of those sick civilians who are their first charge, and in trust for whom they have received the contributions which they dispense. This is a view which has already cropped up at certain hospitals where the question has arisen, and it is one which in some places may become a matter of considerable gravity. We would urge, then, that where beds are offered to the military authorities, they be extra beds, beds not at present needed for their ordinary purposes. Otherwise hard things will be said. It is no uncommon thing to hear people ask what becomes of the patients when ever so many hospitals close their doors in August in order that repairs may be carried out in the "holiday season"—as if even sickness had its close time. The same people will be sure to ask whether some of the appeals for help made by hospitals can possibly be genuine, if whole wards can with propriety be diverted from their normal uses. Let us do good with judgment, and not impulsively.

### The Prescription of Alcohol.

In season and out of season, after the manner of the genus faddist, the teetotalers urge their peculiar crusade. Like the leper of old, who, declining so simple a prescription as a wash in the river Jordan, asked for some great thing, so they decline to preach the simple and wholesome doctrine of temperance, and demand that men should bow before their great fetish of total abstinence. Nor are some of them any too particular about their methods. The last thing that has come to our notice is a suggestion recently made to the medical profession. It is urged that the problem of the inadequate remuneration of medical practitioners is closely bound up with the drink question; that it is impossible to improve matters so long as money continues to be spent in alcoholic drinks by the working classes, and that it is therefore to the interest of the rank and file of the profession to encourage the practice of total abstinence with all their might. As an abstract proposition we agree. But when our own interests are thus made the basis of the suggestion that certain limitations should be laid upon the prescription of alcohol we protest. With the limitations themselves we have nothing to do. They are not materially different from those which are adopted by a large number of medical men as being best for certain of their patients. It is the suggested motive which is objectionable. It cannot be too firmly laid down that the duty of the physician is to the individual patient, and that no other motive than the desire to do the best for that patient should be allowed for a moment to influence the prescription; and when we find the question of our own interests being imported into the problem we cannot but raise our voice against any such misuse of our professional relations. Every medical man has the right, nay, he has the duty, of preaching his faith; but we entirely dissent from the suggestion that a medical man has any right to modify or in any way tamper with the treatment of his patient in deference to the effect of such treatment on those outside, and to suggest, as has lately been done, that if the whole of the medical profession would limit their prescription of alcohol some millions of pounds would be diverted from the liquor trade to more desirable channels, and that in this way the working classes would have more money with which to pay their doctors' bills, is to introduce into a purely medical question matters of self-interest which to our mind are scandalous and abominable. It is urged that no apology is needed for showing to medical men that in taking a course which is to the advantage of the community they are likely to benefit themselves; and with fine contempt for their standard of morality it is further said that the best of men may find it easier to do his duty when he knows that material advantage to himself is likely to follow. To what a low ebb have we have come when such things can be suggested. What has the "advantage of the whole community," or, worse still, what has the "material advantage" to ourselves to do with the question, when what we have to decide is the treatment of a sick man? We pity the patient of the faddist.

### The Fashionable Spittoon!

An ingenious gentleman has hit upon the idea that if the spread of consumption is to be stopped by preventing the scattering of expectoration in public places the first thing to do is to render the pocket spittoon a fashionable appendage. In a sense he is right. Fashion no doubt exercises a mighty sway—may we not almost call it a fifth estate, so putting it next after the press? We are by no means sure that it might not be placed even higher as a power in the land. Hence the force of the ingenious suggestion that all the august persons who have given in their adhesion to the proposed Congress on Tuberculosis should "set the fashion" by beginning at once to carry pocket spittoons and use them openly and unashamed in public places!

